# Development of Mesophasic Microreservoir-Based Transdermal Drug Delivery System of Propranolol

**DOI:** 10.4103/0250-474X.45394

**Published:** 2008

**Authors:** L. K. Omray, S. Kohli, A. J. Khopade, S. Patil, Asmita Gajbhiye, G. P. Agrawal

**Affiliations:** * Guru Ramdas Khalsa Institute of Science and Technology (Pharmacy), Barela, Jabalpur-483 001, India; 1 Government Kalaniketan Polytechnic, Jabalpur-482 001, India; 2 Sun Pharmaceutical Advance Research Center (SPARC), Tandalja, Vadodara-390 020, India; 3 Globus College of Pharmacy, Bhopal-462 045, India; 4 Department of Pharmaceutical Sciences, Dr. Harisingh Gour Universtiy, Sagar-470 003, India

**Keywords:** Liquid crystals, transdermal, drug delivery, *In vitro* evaluation, propranolol

## Abstract

The mesophasic microreservoir comprises lyotrophic liquid crystals. The liquid crystals were prepared of Brij-35, cetosteryl alcohol and propranolol and evaluated for parameters viz. anisotropy, size and size distribution and drug entrapment efficiency. Subsequent to this liquid crystals based transdermal drug delivery system (TDS) was prepared by incorporating liquid crystals in previously prepared matrix based transdermal patch and evaluated for stability studies like temperature, humidity and aging. The system was also studied for tensile strength, moisture content, water vapor transmission, drug content, anisotropy and *In vitro* drug release studies.

Transdermal drug delivery system which releases drug at a zero order rate is now in most cases considered as an ideal system for maintaining constant drug levels. In order to achieve optimal drug therapy and to reduce side effects, many type of transdermal system have been developed till date. The microreservoir and matrix dispersion type drug delivery system offers wide scope in controlling rate of drug delivery. This technology has been successfully utilized in the development of nitroglycerine, oestradiol, clonidine, nicotine and testosterone transdermal patches^1^,^2^.

Present study was aimed at developing a mesophasic (lamellar liquid crystals) microreservoir based TDS of propranolol, a *β* blocker, therapeutically used as an antihypertensive drug. Liquid crystals are mesomorphic or intermediate state between solid crystals and properties characteristic of solids and liquids, *e.g.* possesses both structural ordering and mobility; when viewed under crossed nicol prism of polarizing microscope, intense color bands and birefringence are seen. The liquid crystalline phase is thermodynamically stable and represents a state of incomplete melting^3^.

During the past decade, there has been great interest in liquid crystalline systems as drug delivery systems in the field of pharmacy. The reasons for this interest includes the extensive similarity of these colloid systems to those in living organism and their advantageous properties over those of traditional semisolid dermal dosage forms. Their formation is explained by the spatial organization of aggregates of nonionic surfactant molecules, at lower and higher concentrations. These lyotropic mesophases are usually formed from water and one or two surfactant and possibly co surfactants as very definite proportions with low energy input or by means of spontaneous structural organization. Their production is relatively simple and energy saving. They are thermodynamically stable and can be stored unchanged for long periods of time without phase separation. Depending on the concentration of the solvent (generally water or an aqueous solution) and on the polarity of the solvated mesogen, these systems can undergo various phase transition. As a consequence, their consistency can be changed systematically^4^,^5^. These drug bearing liquid crystals were utilized for the fabrication of transdermal patch. The objective was to effectively deliver propranolol across the skin.

## MATERIALS AND METHODS

Propranolol hydrochloride was obtained from Lupin Lab. Pvt. Ltd.; Aurangabad, India, Cetosteryl alcohol was procured from Central Drug House Pvt. Ltd., Mumbai, Brij-35 and hydrogel forming polymers have been purchased from E. Merck, Mumbai and were used without further purification. All other ingredients unless otherwise specified were of analytical reagent grade. Double distilled water was used throughout the experiment.

### Conversion of propranolol hydrochloride to propranolol:

Propranolol hydrochloride (5 g) was dissolved in 100 ml distilled water. This solution was made alkaline by adding gradually 5% w/v aqueous solution of sodium hydroxide. A white precipitate of propranolol was formed which was filtered and washed on Whatman filter paper No1, until the washings were free from any traces of chloride^6^.

### Preparation and characterization of hydrogel based free film:

Various composition of film forming polymer were dissolved in a sufficient quantity of distilled water. In a separate test tube 1% v/v of PEG-400 and 3% v/v of propylene glycol (percentage according to the total quantity of film forming solution) were gently mixed to avoid the formation of froth. The resulting gel like solution of polymers and plasticizers were poured on siliconised glass mould and dried in an oven at 50°. The films were qualitatively characterized for their acceptability and stored in a polyethylene bag at room temperature separately^7^,^8^.

### Preparation and characterization of simple lyotrpic liquid crystals:

Liquid crystals (LC) were prepared by the method reported by Eccleston and Beattie^9^ with a slight modification. The cetosteryl alcohol (2.5 g) and Brij-35 (1.1 ml) were melted together and 30 ml of double distilled water, which had been previously boiled to remove air was added at approximately the same temperature (80°) followed by cooling slowly whilst mixing with a Silverson homogenizer. The homogenizer speed was controlled throughout to avoid incorporation of air. The 300 mg of propranolol was incorporated in the mixture of cetosteryl alcohol and Brij-35. The liquid crystals were stored at 25° in an air tight glass container.

### Optical microscopy:

Liquid crystals were examined using the polarized light microscope (Nikon HFX Labohot) in bright field and between crossed polars. The system was examined immediately after preparation and at frequent intervals over two month storage period.

### Size and size distribution:

Liquid crystals were mounted on a glass slide and viewed under the polarized microscope under magnification of X40 to determine size and size distribution using ocular and stage micrometer.

### Entrapment efficiency:

Entrapment efficiency is the percent of total drug entrapped in liquid crystals or molar fraction *i.e.* the ratio of entrapped drug and lipid. The unentrapped free drug from liquid crystals dispersion was removed by placing the 5 g of liquid crystals dispersion in dialysis tube (Spactrapore, membrane size 5000-7000 molecular weight cut off), thickness 25 μm, (Los Angles, USA) and dialyzing exhaustively against distilled water. The free drug separated in distilled water was analyzed spectrophotometrically at 290 nm using a Shimadzu double beam spectrophotometer.

### Preparation and characterization of mesophasic microreservoir based transdermal system (MMTS):

MMTS was prepared using selected polymer solution, film code L-10, and the simple lyotropic liquid crystals in a ratio of 2:1. These two were mixed in a Silversion homogenizer at constant rate to avoid the entrapment of air; the resulting solution was poured in a glass mould and dried in an oven at 45°. The films were stored in polyethylene bag at room temperature.

### Optical microscopy:

MMTS was examined using the polarized light microscope (Nikon HFX Labophot) in bright field and between crossed polar to study the anisotropy of film and uniform distribution of liquid crystals.

### Drug content:

The MMTS film was cut into pieces of 1 cm^2^ which was further fragmented into pieces. The drug from the polymer film was extracted in chloroform. Polymeric film was removed from solution by filtration. The chloroform was evaporated and the residue was dissolved in 5 ml of methanol and volume made up to 100 ml in volumetric flask with distilled water. One ml of this solution was further diluted to 10 ml with distilled water and absorbance was measured against distilled water as blank at 290 nm^10^.

### Thickness and Tensile strength:

The meter dial gauge (Mercer, USA) was used for the determination of film thickness having a least count of 0.002 mm. The tensile strength (TS) of the film was determined by fixing both the ends of the film between the adhesive tapes to give support while fixing between two iron plates. A small hole was made in the adhesive tape near the iron plates in which a hook was inserted fitted with a small pan. Weights were gradually added to the pan to increase the driving force till the film was broken. Tensile strength was determined by the formula, TS = (Break force/a.b)×(1+dL/L)^7^,^11^, where, L, a and b are length, width and thickness of the test strip respectively and dL is the elongation at break.

### Water vapor transmission rate (WVT) and Moisture content (MC):

The WVT was determined by the method reported by Kanig and Goodman^12^ at 25±2° and 63% RH. WVT rates were calculated by the formula of Kanig and Goodman and results are recorded in Table 2. WVT = Amount of moisture transmitted/Areaxtime. The weighed film samples were kept in an oven at a temperature of 100×2° for drying for six hour. The percent moisture content (MC) was calculated as % MC = weight of water in sample%100/weight of dry sample.

**TABLE 2:** SIZE AND SIZE DISTRIBUTION OF SIMPLE LC

Range (μm)	Mean size (μm) X	Frequency (n)	Xn
0-5	2.5	68	170
6-10	8.0	126	1008
11-12	13.0	83	1079
16-20	19.0	16	304
21-25	23.0	7	161
	Total	300	2722

All values are expressed as mean of three observations; Mean size of liquid crystals LC, 9.073 μm.

### *In vitro* drug release studies:

The *in vitro* release of propranolol from liquid crystals and MMTS was studied using locally fabricated Franz diffusion type cell^13^,^14^. A commercial semipermeable membrane was employed in the study as the permeation barrier (Spactrapore, membrane size 5000-7000 molecular weight cut off), thickness 25 μm, (Los Angle, USA). The semipermeable membrane (2.5 cm^2^) was mounted on the receptor compartment of the diffusion cell and product approximately equivalent to 5 mg of the drug was applied. The receptor compartment contained 30 ml of the isotonic buffer (pH-6) solution. Samples of 5 ml were withdrawn at a time interval of two hour and the same was replaced with 5 ml of the fresh media solution in order to maintain the sink condition. The withdrawn samples were diluted appropriately with isotonic buffer (pH-6) solution. The samples were analyzed spectrophotometrically for drug content at 290 nm using double beam Simazdu spectrophotometer^10^.

### Stability studies:

The stability studies of the selected product were performed by measuring tensile strength, percent moisture content, water vapor transmission, drug content and microscopy. MMTS was packed in aluminium foil and then stored in polyethylene bag before conducting stability studies. For effect of temperature, the selected MMTS were kept in desiccators (51% RH) and stored at 37, 45 and 55° for 30 d and the samples were evaluated for various parameters. For effect of humidity, the selected MMTS were kept in desiccators maintained at different relative humidities^15^ (35, 51 and 79% RH) for 30 d at room temperature and evaluated for different parameters. The atmospheres of 35, 51 and 79% RH were produced by taking the saturated solution of calcium chloride, magnesium nitrate and ammonium chloride respectively in separate desiccators. For effect of aging, the MMTS were kept in desiccators at 51% RH and room temperature for 30 and 60 d and studied for various parameters.

## RESULTS AND DISCUSSION

Conversion of hydrochloride salt of drug to free base increases the lipophilicity of the permeant molecules. The reported value of the logP (partition coefficient) of propranolol free base is 1.2 and its pKa is 9.5, which indicates the relative affinity of the free propranolol base toward the nonpolar phase. Polar nature and low skin permeability of the propranolol hydrochloride salt make it unsuitable for transdermal application. Because of high permeability, favorable logP value and greater pKa, free base was selected for the development of transdermal drug delivery system^16^.

MMTS of propranolol was fabricated using glass mould. The study was carried out in three steps. The first step involves the preparation and evaluation of matrix system, the next step was preparation and evaluation of liquid crystals and the last step was preparation and evaluation of MMTS.

Hydrogel based free film were prepared by the technique described by Kuriya *et al.*^6^,^12^. The plasticizer concentration was optimized for smooth and clear film; films were evaluated for thickness, tensile strength, moisture content, water vapor transmission and physical appearance. The film L-10 was selected on the basis of various parameters such as for the preparation of MMTS. The thickness, tensile strength and moisture content maintain the integrity, shape and composition of film. WVT directly influence the absorption of drug by affecting hydration of skin. Higher WVT remove water from stratum corneum and low WVT keeps skin hydrated hence enhanced drug permeation through skin.

Tensile strength of various films was found in the range of 0.82 to 1.32 Kg/mm^2^, which is good characteristic to handle the film. Moisture is a part of film composition, which affects WVT, structural characteristic of film and hydration of skin. MC was found to vary from 6.21% to 7.23%; the difference may be attributed due to the nature of polymer. The compositions of hydrogel forming polymer and their evaluation data are shown in Table 1.

**TABLE 1:** THE EVALUATION DATA OF HYDROGEL FREE FILMS

Film Code	Polymer Ratio and % mixture*	Thickness mm	WVT×10^−4^ g/h/cm^2^	Tensile strength Kg/mm^2^	% moisture content	Physical appearance
L-1	HPMC 5%	0.38	4.41	0.82	7.22	S,C,A
L-2	PVA 5%	0.40	3.92	1.11	6.95	S,C,T
L-3	HPMC:PVA 20:80, 5%	0.38	4.42	1.10	7.12	S,C,T
L-4	HPMC:PVA 60:40, 5%	0.40	4.41	0.96	6.92	S,C
L-5	HPMC:PVA 80:20, 5%	0.40	4.02	0.81	6.98	S,C,W
L-6	HPMC:PVP 80:20, 5%	0.38	4.39	0.82	7.23	S,C
L-7	HPMC:PVP 50:50, 5%	0.38	4.36	0.78	7.11	S,C
L-8	HPMC:PVP 30:70, 5%	0.36	4.28	0.88	7.20	S,W,C
L-9	PVA:PVP 50:50, 6%	0.46	3.99	0.96	6.42	S,C,F
L-10	PVA:PVP 80:20, 6%	0.48	3.91	1.32	6.21	S,C,F
L-11	PVA:PVP 60:40, 6%	0.46	3.98	1.11	6.38	S,C,F

All values are expressed as mean of three observations; T, Toughness; S, Smooth; C, Clear; A, Entrapment of Air; W, Wrinkled; HPMC, Hydroxypropylmethylcellulose; PVP, Polyvinylpyrolidone; PVA, Polyvinyl alcohol; F, fine;

* W/V, weight / volume

Preparation and evaluation of liquid crystals: Liquid crystals are mesomorphic or intermediate state between solid crystal and isotropic liquid. They are bilayer vesicular system exhibiting anisotropy (having optical direction) and properties characteristic of solids and liquids *e.g.* possesses both structural ordering and mobility, when viewed under crossed nicol prism of a polarizing microscope intense color bands and birefrigerence are seen. The liquid crystalline phase is thermodynamically stable and represents a state of incomplete melting. The drug bearing liquid crystals was utilized for the fabrication of transdermal patch.

Simple lyotropic liquid crystals were prepared from Brij-35 and evaluated for anisotropy, size and size distribution and drug entrapment efficiency. Anisotropy and birefrigerence of the system in polarized light microscope proved the presence of liquid crystals. The blank and drug loaded liquid crystals did not show any optical change when viewed under polarized light microscopy. The particle size and size distribution was found in the range of 0.00 to 25.00 μm and mean size was 9.02 μm. The entrapment efficiency was found to be 94±2.5%. The higher entrapment efficiency may be due to the higher drug solubility in lipophilic solvent and incorporation of only such amount of water which was required for the formation of liquid crystals. The calculation of average particle size and size distribution are shown in Table 2.

**Fig. 1:**
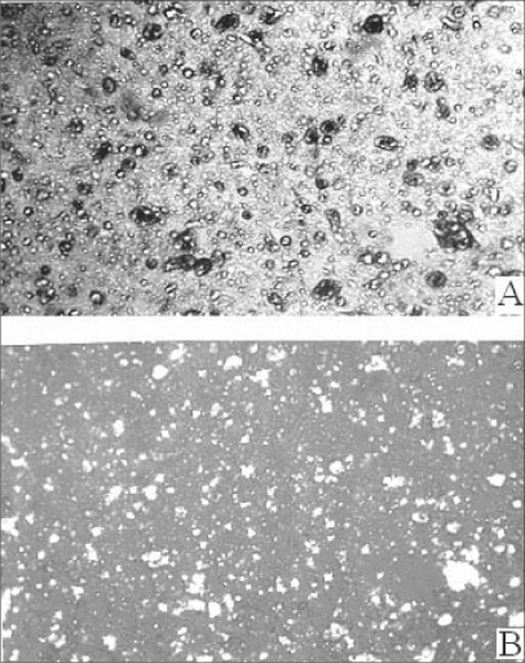
Liquid crystalline structure of MMTS Liquid crystalline structure of mesophasic microreservoir-based transdermal system MMTS (X40) under (A) Plain microscope (B) Cross polarizer.

MMTS was prepared and evaluated using selected free film forming polymer composition L-10 as shown in table no. 1 and the drug bearing liquid crystals. This system was evaluated and data is shown in Table 3. The anisotropy of MMTS proved the integrity of liquid crystals within polymeric film, their distribution in the film was found to be uniform. The microphotographs of MMTS under plain and cross polarized microscope are shown in fig. 1a and 1b respectively.

**TABLE 3:** CHARACTERIZATION OF MMTS

Parameters	Observation
Film thickness (mm)	0.42
WVT × 10 g/h/cm^2^	3.54
Tensile strength Kg/mm^2^	1.16
Moisture content %	7.86

All values are expressed as mean of three observations. MMTS, mesophasic microreservoir-based transdermal system

*In vitro* release studies of drug from liquid crystals and liquid crystals dispersed films were performed. The *In vitro* release studies were performed using isotonic buffer solution (pH 6.0) as a receptor fluid owing to its simulation with the physiological condition of the mammalian skin. The pH of the mammalian skin varies in acidic range from 4.5 to 6.0 and the acidity is due to the presence of amphoteric amino acid, lactic acid and fatty acids in the secretion of sebaceous glands^17^. It is essential to estimate *In vitro* drug release in buffer solution of these pH ranges. The solubility of the propranolol free base is sufficient at 25° in buffer solution at pH 6.0 to provide sink effect during *In vitro* diffusion study.

The absorption of drug through the skin occurs by passive diffusion. The rate of drug transport across the stratum conium (SC) follows Fick's law of diffusion: J= dM/Sdt=DΔcK/h, where dM/Sdt (J) is the steady-state flux across the SC, D is the diffusion coefficient or diffusivity of drug molecules, Δc is the drug concentration gradient across SC layer, K is the partition coefficient of the drug between skin and formulation medium and h is the SC thickness. It can be summarized as the rate of drug absorption directly proportional to its oil/water partition coefficient, its concentration in the formulation vehicle, and the surface area of the skin to which it is exposed; it is inversely proportional to the SC thickness.

According to the Higuchi equation, plot of the cumulative amount of drug released per unit area (μg/cm^2^) against the square root of time yields a straight line; the slop of the regression line represents the release rate. The Higuchi equation is as follows Q= (2ADCs t)^½^, where Q is the cumulative amount of drug released per area of the matrix, A is the total drug concentration in the matrix, dissolved and undissolved, D is the diffusion coefficient of the drug in the matrix, Cs is the solubility or saturation concentration of the drug in the matrix and t is the time. The Higuchi equation on the basis of rate of drug release is as follows dQ/dt=k/t^½^, where k is the release constant. Plot of dQ/dt against the reciprocal of the square root of time leads to a straight line^18^.

**Fig. 2:**
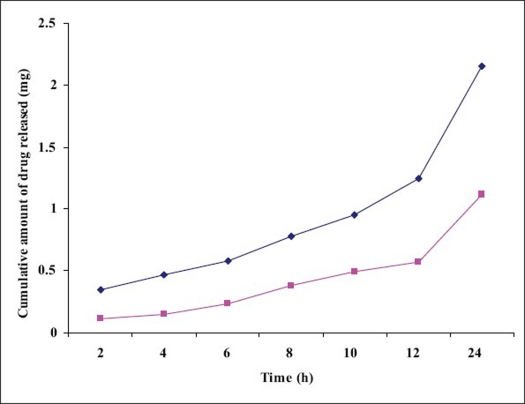
*In vitro* release studies of propranolol from LC and MMTS *In vitro* release studies of propranolol from liquid crystals (LC, -◆-) and mesophasic microreservoir-based transdermal system (MMTS, -■-)

*In vitro* release studies of the developed formulations are represented in fig. 2. A comparison of the release graphs of the two formulations (LC and MMTS) shows controlled release of the propranolol for 24 h. This could be due to the fact that the drug available in a form of solution, drug inside liquid crystals and these liquid crystals releases the drug in a controlled release manner^19^. In order to get a better insight into the mechanisms underlying the controlled release of propranolol from the transdermal systems, the release kinetics of propranolol was investigated. The result in fig. 2 indicate that the transdermal systems (LC and MMTS), release propranolol gradually throughout the 24 h study, this could be due to controlled release of drug from LC and controlled release and polymer matrix diffusion controlled process from the MMTS. The cumulative amount of propranolol released from MMTS was about 1.12 mg in 24 h. The amount released from MMTS was less than that released through LC alone (2.15 mg/24h). This could be attributed to uniform distribution of the drug in polymeric film and MMTS also face another diffusion step *i. e.* polymer matrix diffusion controlled process. The *In vitro* release data was treated with Higuchi's kinetic diffusion equation. The release however followed Q α t^½^ equation, showing the Higuchi control on release rate. This may be due to the composition of transdermal system.

MMTS was packed in aluminum foils and were subjected to exaggerated stability studies *e.g.* temperature, humidity and aging. The stability studies data are summarized in Table 4. It was found that all the parameters did not show any sharp change in the film, which indicate that the liquid crystals were stable and compatible in these systems even after the long period of storage. Hence it can be concluded that the mesophasic micro-reservoir based transdermal systems are easy to prepare, stable and safe and can be produced commercially for drugs employed for effective treatment of hypertension, cardiac arrhythmias and angina pectoris.

**TABLE 4:** EFFECT OF TEMPERATURE, HUMIDITY AND AGING ON THE STABILITY OF MMTS

Parameters	Initial value	Stability							
		
			Temperature°			RH		Aging (d)	
		37	45	55	35	51	79	30	60
TS Kg/mm^2^	1.16	1.14	1.08	0.98	1.15	1.12	0.99	1.15	1.17
% MC	7.86	7.48	7.11	6.93	7.83	7.92	8.63	7.89	7.84
WVT g/h/cm^2^	3.54	3.56	3.58	3.49	3.57	3.62	3.99	3.56	3.57
M	A	A	A	A	A	A	A	A	A
DC mg/cm^2^	2.71	2.68	2.59	2.46	2.69	2.70	2.66	2.68	2.65

All values are expressed as mean of three observations; TS, Tensile strength;% MC, Percent moisture Content; WVT, Water vapor transmission; M, Microscopy; A, Anisotropy; RH, Percent relative humidity; DC, drug content and MMTS, mesophasic microreservoir-based transdermal system
